# Joining the dots: neurobiological links in a functional analysis of depression

**DOI:** 10.1186/1744-9081-6-73

**Published:** 2010-12-11

**Authors:** Christopher F Sharpley, Vicki Bitsika

**Affiliations:** 1 Brain-Behaviour Research Group, University of New England, New South Wales, Australia; 2 Brain-Behaviour Research Group, Bond University, Queensland, Australia

## Abstract

Depression is one of the major contributors to the Total Disease Burden and afflicts about one-sixth of Western populations. One of the most effective treatments for depression focuses upon analysis of causal chains in overt behaviour, but does not include brain-related phenomena as steps along these causal pathways. Recent research findings regarding the neurobiological concomitants of depressive behaviour suggest a sequence of structural and functional alterations to the brain which may also produce a beneficial outcome for the depressed individual--that of adaptive withdrawal from uncontrollable aversive stressors. Linking these brain-based explanations to models of observable contingencies for depressive behaviour can provide a comprehensive explanation of how depressive behaviour occurs and why it persists in many patients.

## Background

Principally via its harmful effects on health, relationships and cognitive performance [1-4], depression is the principal contributor to the Total Disease Burden [5] and predicted to become the second leading cause of mental illness by 2020 [6,7]. As an indication of the significance of this problem, it has been shown that depression poses as great a risk for mortality as does smoking, even when controlling for blood pressure, alcohol intake, cholesterol and social status [8]. The incidence of having a major depressive episode during one's lifetime is 17% in the USA and 13% in Europe [9-11], suggesting that nearly one in five people may suffer this disorder. Together, these data argue for the urgency of research into effective treatments for depression, particularly those that are based upon explanations of depression that encompass both externally-observable behaviour and also brain function.

## Treatment of depression

Both pharmacological and psychological treatments have been shown to be effective with various populations of depressed persons [12,13], with some data suggesting a reciprocal relationship between these two approaches [14,15]. One form of psychological treatment that has a strong evidence basis and which is relevant to a discussion of the links between brain and behaviour in explaining the 'causality' of depression is that which is commonly referred to as "Behavioural Functional Analysis". This process underlies many successful psychological treatments [e.g., 16, 17-22], and is characterised by a focus upon the *purpose *or *outcome *of behaviour for the organism exhibiting it [21], information that is acquired by examining the antecedents and consequences of a particular behaviour and determining the links between these two aspects. As well as being successful with mild to moderate depression, there are also data which support the use of some of these functional analytic approaches with Major Depressive Disorder (MDD) [23], indicating that this form of psychotherapy may be at least as effective as antidepressant medication for reducing the symptoms of acute depression [24-26].

This process of analysing the antecedents and consequences of overt behaviours as causal vectors for depression is the central theme of Behavioural Functional Analysis, and may reveal the outcomes of depressive behaviour for the individual exhibiting it [27,28]. For example, depressive behaviour may (unwittingly) be negatively reinforced by avoidance of, or escape from, threats, and positively reinforced by social attention and help from peers, relatives or (in the case of humans) professionals who focus upon the depressed individual's suffering and attempt to alleviate it by various means [29]. These consequences for depressive behaviour may provide powerful contingencies for maintenance of the depressive symptomatology, at least for a period of time. As noted 150 years ago by Charles Darwin (p. 51):

Pain or suffering of any kind, if long continued, causes depression and lessens the power of action; yet, it is well adapted to make a creature guard itself against any great or sudden evil" [30].

Thus, depressive behaviour may provide a (temporary) benefit for individuals when they encounter uncontrollably aversive environments, and this model is congruent with the hypothesis advanced several decades ago by Ferster [31], Dougher and Hackbert [32] and most recently elaborated by Kanter, Busch, Weeks, and Landes [33] that depressive symptomatology falls within the spectrum of withdrawal from contact with an uncontrollably aversive environment [29]. Although the "adaptive" nature of that withdrawal may become maladaptive over time, the initial function of the depressive behaviours is adaptive for the person exhibiting it.

This perspective is parallel to the notion of "sickness behaviour" in humans and other animals as postulated by Dantzer, O'Connor et al. [34], who identified symptoms such as feeling feverish and nauseous, experiencing a lack of appetite, sleep disturbance, loss of interest in social and physical environments, anhedonia, and being easily fatigued, as normal responses to infection and which are triggered by pro-inflammatory cytokines such as IL-1β and TNF-α. That is, as well as coordinating the local inflammatory response to infection, IL-1β and TNF-α also act on receptors spread throughout several regions of the brain (e.g., dentate gyrus, pyramidal cell layers of the hippocampus, anterior pituitary gland, hypothalamus and others [35,36]) to cause these sickness behaviours. In terms of their function, these sickness behaviours help infected individuals cope with their illness by changing their perceptions of their state and their reactions to it.

However, in some cases, those sickness behaviours may be so severe as to constitute the necessary symptomatology for a diagnosis of MDD by way of the social withdrawal that characterizes cytokine-induced sickness behaviour and which also underlies all depressive symptomatology [29]. Dantzer, et al. reported that administration of IL-1β or TNF-α to rats induced sickness behaviour such as withdrawal from social activity, reduced motor behaviour, reduced water and food intake, increased fatigue, altered cognition and changed sleep patterns. The rats "stay in a corner of their cage in hunched posture" (p. 48), reminiscent of the behaviour of many humans with MDD [37]. When applied to the specific MDD symptoms of sadness, anhedonia, sleep disturbance, appetite change and cognitive impairment, these symptoms may be seen to represent a biological response pattern that translates into the psychological behaviours of low self-esteem, hopelessness and helplessness. Although these may be unpleasant, this withdrawal from aversive stimuli and environments that characterises depression has been described as "adaptive" because it also reduces the quantum of noxious stimuli to which the organism is exposed [38,39]. An important aspect of the mechanism underlying this withdrawal is the conviction on the part of depressive individuals that they have no real control over the unpleasant experience that is occurring to them [38,39] and therefore are left with a single response that will reduce distress---withdrawal from the environment that is causing it. In this way (and as indicated by Darwin), depression may perform a valuable function for the depressed individual [40,41] by reducing unpleasantness in the only way possible at that time.

However, psychological treatments for depression that use Functional Analysis (to at least some extent) have focussed almost exclusively upon the readily observable (i.e., overt) environmental antecedents and consequences for depressive behaviour. Explanations based upon those data alone are incomplete because they do not describe the less-easily observed neurobiological links between the organism experiencing the genetic and environmental stimuli and the later emergence of depressive behaviour. A description of those neurobiological links to depression would not only provide a more complete and logical description of the development of depressive behaviour as a typical response to overpowering and aversive environmental stress, it could also inform treatment of depression.

From a biological standpoint, while some of the antecedents for depression are genetic [42-45], one of the strongest predictors of depression is stress in the form of excessive demanding challenges across a range of areas [46]. Stress acts through a variety of physiological causal pathways which may undermine an individual's ability to withstand it [47,48] so that, over prolonged time, depressive symptoms occur. Those causal pathways include not only the *form *of the externally-observable behaviour, the *function *of that behaviour (in terms of outcomes gained by the organism exhibiting it), but also a consideration of how brain responses *act in concert *with those behaviours to produce an overall valued outcome for the depressed individual.

Therefore, this paper addresses the issue of linking depressive behaviour with brain function and (a) describes the neurobiological steps between onset of an environmental stressor and the later development of depressive behaviour, (b) explains how these steps function for the benefit of the depressed individual, and (c) raises some implications for treatment options.

## The neurobiology of depression

There are several subtypes of depression, but the most common is unipolar depression, characterised by the presence of anhedonia and general sadness, plus a range of other cognitive, physical or emotional symptoms [37]. Some of the brain-based aspects of depression link with its psychological symptoms, indicating that this disorder needs to be considered from a unified perspective. For example, depressed patients can exhibit: depressed mood, which may be associated with dysfunction of the activity of the left dorsolateral prefrontal cortex [49]; excessive guilt and hopelessness, which is linked with dysfunction of the hypothalamic-pituitary-adrenal (HPA) axis [50-53]; psychomotor agitation, loss of weight and sleep problems, perhaps reflecting abnormalities in the function of the thyroid axis [54]; and disturbances to Random Eye-Movement sleep, commonly caused by changes to circadian rhythms [55].

Most brain functions rely upon the presence of various neurotransmitters. Reduced availability of those neurotransmitters causes impaired cognitive performance and may lead to depression [56]. This process has been described as the "monoamine hypothesis" because it focuses upon the reduced presence of monoamine neurotransmitters in depressed persons, a state that is brought about via the degrading actions of monoamine oxidases in the synaptic cleft [56]. By applying anti-oxidase agents in the form of antidepressant medication, levels of monoamine neurotransmitters may be restored [57,58]. Serotonin, noradrenalin and dopamine are particularly important monoamine neurotransmitters that have been inculcated in depression [56]. Serotonin (or 5-HT) has the largest cohesive neurotransmitter system in the brain and innervates all brain areas [59]. Changes to serotonin have been shown to influence the core behavioural and somatic functions that underlie depression, such as appetite, sleep, sex, pain response, body temperature and circadian rhythm [60], and post-mortem studies indicate lowered levels of serotonin in the brains of depressed patients [61-63]. Noradrenalin is also a major neurotransmitter, with noradrenergic neurons spreading from the brain stem to almost all brain areas and modulating the function of the prefrontal cortex (which uses working memory to regulate behaviour and attention), as well as having an important role in the acquisition of emotionally-arousing memories [59]. Dopamine modulates activity in brain areas involved with reward and motivation, working memory and attention [64], and reduced dopamine has also been inculcated in the development of depression [65]. As may be expected, serotonin, noradrenalin and dopamine are primary targets for pharmacological treatments for depression.

Genetic bases for the reduced functionality of serotonin [66] and dopamine [67] have been established. However, these genetic influences do not operate in a vacuum, and the interaction of environmental stress with genes has been shown to be reciprocal [68]. Adolescent depression develops via the experience of aversive environmental events and chronic stress during childhood, acting through prolonged HPA activation as indicated by elevated cortisol [69,70] in those individuals who carry particular genes that are up-regulated by the adverse experiences of childhood. For example, early life adversity decreases expression by the gene for Brain-Derived Neurotrophic Factor, which mediates neural plasticity in the prefrontal cortex (PFC) and hippocampus [71]. Interactions between environmental events and genetic predisposition to depression reduce neurogenesis in the hippocampus (an area which is centrally involved in the storage and accessing of memories that underlie emotional expression), thereby contributing to lowered mood and depression [46]. Taken together, these data provide support for the "Diasthesis-Stress hypothesis" [72,73], under which interactions between the environment and genes lead to associated neurobiological and structural changes in the brain that are linked to depression [74-76]. The major pathway through which environmental adversity leads to gene-linked changes in neurotransmitter function, brain structure and thence depression, is the HPA axis.

## How the HPA axis links genes, environmental stress and depressive behaviour

Hyperesponsivity of the HPA axis under stressful environmental conditions has been associated with depression in several studies [e.g., [50-53]]. When highly activated, the HPA axis produces elevated serum cortisol, leading to hypercortisolaemia, which can cause changes to those regions of the brain that are associated with cognitive processing of how threatening a particular environmental demand may be to the individual and how that threat might be dealt with (i.e., PFC and hippocampus), plus those regions which are centrally associated with intensifying emotional responses (i.e., amygdala) [77]. Specifically, these hypercortisolaemic effects include dendritic branching and neurogenesis which produce *increases *in the volume of the amygdala [78], and cell apoptosis which *decreases *the volumes of the PFC [79] and hippocampus [80]. There are also alterations to the connectivity of those regions of the brain, for example between the amygdala and the PFC [81] and between the amygdala and the hippocampus [82]. These organic changes to the brain have been linked to the eventual development of those withdrawal behaviours that underlie depressive symptomatology [29,33]. Those brain region and interconnectivity changes have been shown to be a direct outcome of hypercortisolaemia, and this pathway between environmental stressor-induced hyperactivation of the HPA axis and depression represents the neurobiological link between those stressors and the depressive symptomatology that is described in behavioural accounts of depression.

The accepted symptomatologies of depression (i.e., DSM, ICD) highlight the specific presence of apathy and anhedonia, which are localised to the reward circuits in the limbic system that are heavily reliant on dopamine [83], leading Nestler, et al. [76] to call that limbic reward system the "neural circuitry of depression" (p. 16). For example, amygdala blood flow increases, but PFC blood flow decreases, in depressed patients compared to non-depressed controls [84], supporting the finding that increases in amygdala function are related to depressive state [85]. In addition to this emotional aspect of depression, and because the PFC is a key area for motivation to change or adapt to demanding environments, damage to the PFC also impairs these resilience-oriented abilities, and apathy may develop. As mentioned above, apathy is the central symptom of depression [86], and prevents depressed individuals from taking action to reduce their unhappiness via more productive problem-solving and lifestyle strategies [37]. Thus, impairment of the PFC is directly linked to this key symptom of depression [87].

These structural changes to the amygdala, PFC and hippocampus also induce elevated anxiety behaviour, which may then instigate even greater elevation of hypothalamic responses to stress. When the individual is threatened, the activation of the amygdala stimulates the hypothalamus for fight-or-flight responses. By contrast, the hippocampus has an inhibitory effect upon the hypothalamus [88], principally via the involvement of its ventral region in inhibition of the sympathetic nervous system (SNS) and subsequent reduction of anxiety behaviours [89]. While anxiety may occur alone, it is often comorbid with depression [90], and there is also major overlap in the symptomatology for both disorders [91]. These links between hippocampal inhibition of anxiety and the amygdaloid activation of fear via hypothalamic neuroendocrine secretions may represent a "balance" of efforts by these two regions that has the effect of allowing the individual to manage threat effectively [92]. When that balance is disturbed by alterations to the hippocampus and amygdala (and their comparative influence on the hypothalamus), disturbances in mood follow, contributing to the likelihood of clinical depression.

These findings suggest a model of depression in which a circuit including the PFC, amygdala and hippocampus influences not only mood regulation, but also learning and contextual memory processes [88]. Specifically, during major depression the ventromedial prefrontal cortex (VMPFC) and lateral orbital prefrontal cortex (LOPFC) are hyperactivated and the dorsolateral prefrontal cortex (DPFC) is hypoactivated [93,94]. These changes are consistent with the symptoms of major depression because hyperactivation of the VMPFC is associated with sensitivity to pain, anxiety, depressive rumination and tension, while hypoactivity of the DPFC is associated with psychomotor retardation, apathy, and deficits in working memory and attention [88]. Studies of the connectivity between these areas have also suggested that decreased communication between the amygdala and anterior cingulate complex (ACC) occurs in major depression [95]. Because the ACC acts to inhibit emotional regulation [89], decreases in communication between it and the amygdala could be involved in the irregularity of mood that is another symptom of depression [88].

These details are important in forming a causal chain that links environmental stressors to depressive behaviour because, when connectivity is disrupted between the integrative and executive functions of the lateral orbital PFC, rostral PFC, medial PFC, dorsolateral PFC and dorsal ACC on one hand, and the emotional/visceral functions of the ventral ACC and ventral medial PFC, plus the hippocampus, amygdala and nucleus accumbens on the other hand, the brain undergoes a decrease in regulatory feedback from the former "rational" regions to the latter "emotional" regions [92]. This allows the latter to dominate control of the hypothalamus and consequent neuroendocrine activity, leading to further stress responses and SNS dominance [77], plus increases in fear and consequent withdrawal by the fearful/depressed individual from their physical and social environment [29,33].

## Explaining functional outcomes of depression from a behavioural-neurobiological perspective

As indicated above, the amygdala enlarges and the PFC and hippocampus shrink either before or during the initial stages of depression, producing a more emotion-oriented response pattern in the depressed individual, principally via elevated activation of the HPA axis, hypercortisolaemia, increased hypothalamic activity and downstream increases in SNS activity. Although this emotionality might initially appear to be at odds with the relatively "flat" affect that is symptomatic of depression, it may more easily fit within the early stage of depression, when the patient is faced with uncontrollable aversive environmental events. When faced with that kind of insurmountable environmental stressor, the relative usefulness of completely rational problem-solving strategies is probably low, simply because of the powerlessness experienced by the depressed individual when they consider their situation rationally. That is, compared to emotional responses that may enhance the individual's likelihood of (even by chance) finding a strategy out of the uncontrollable aversive situation they are in, rational thinking may be less likely to be selected by environmental pressures. In addition, these heightened emotional responses in the face of uncontrollable aversive stressors may result in the depressed individual becoming extremely frightened and even behaviourally "freezing" or withdrawing, and thereby avoiding further unwanted aversive outcomes. This phenomenon of "freezing" is common among mammals as a response to a predator and has a functional value in that it shows submission and hence may cause the predator to cease attacking [96,97]. Freezing may also perfor a valuable function because many predators' actions are triggered by movement, and they may lose interest when the prey remains motionless for some time, thus conveying a survival advantage upon the frozen prey [98]. Additionally, some predators do not eat dead meat and may refrain from eating prey that appears to be dead (i.e., immobile) [99]. Similar freezing behaviour has been reported in humans when faced with rape [100] and airline disasters [101], and has been described as "an evolutionary-based fear response" [102].

However, although several authors have described depression as "evolutionary" in that it has evolved to perform the functions of (1) withdrawing from uncontrollable aversive stressors and allowing the depressed individual time to analyse the stressful situation they find themselves in, and (2) eliciting assistance from partners and close others [102,103], this model is not without its critics [104,105] and may also be misnamed on biological grounds because depressed individuals do not possess any visible reproductive advantage, nor do their offspring [106]. More accurately, depression might be considered to serve the *immediate *adaptive function of withdrawal in the face of uncontrollable aversive environmental stressors, i.e., it performs a function for the depressed person *at the time of the stressor*. That function may include reduction in the intensity of the environmental stressor's effect upon them, provision of time for reflection on future responses to the stressor, allowing time for the stressor to pass, and as a passive invitation to others to take some responsibility for the depressed individual's welfare. In more modern times, depression may also serve the individual by providing intense nurturing in the form of psychotherapy [107], reduction of the organic unpleasantness associated with depression via antidepressant medication [108], reduction of workload via formal sick-leave [109], and wider social support from family and friends.

When viewed from this perspective, the neurobiological responses of: (i) elevated HPA axis activation in response to overwhelming stressors, followed by consequent hypercortisolaemia, which causes (ii) structural alterations to the amygdala (enlargement) and PFC and hippocampus (shrinkage) that lead to (iii) amygdala dominance of the hypothalamus, causing (iv) more emotional and SNS-focussed behavioural activity patterns, can be understood as a chain of neuro-behavioural responses to threat that may possess some immediate benefit for the individual exhibiting them. Concomitantly to these adjustments to uncontrollable stress, emotional responses to such adversity engender some typical physiological responses including crying [37], an activity which secretes endorphins, thereby providing some relief from the depression, however transient [110]. Other physiological responses observed during depression include sleepiness (which provides respite from awareness of the aversive environmental stimuli), and compensatory behaviours such as over-eating (which may be distracting and/or pleasant) [37,107]. Coupled with apathy, reduced interest or pleasure in activities relating to the stressful environment, and overall social and behavioural withdrawal, these observable phenomena comprise the major symptomatology of MDD.

## Using a behavioural-brain model in therapy: Implications for treatment

As well as explaining the link between aversive environmental stressors, withdrawal (depressive) behaviour and escape, plus access to valued environmental consequences via functional analysis as described above, the range of behavioural therapies share the principal effective components of (i) building rapport between therapist and patient [111,112], and (ii) behavioural homework activities which are recommended by therapists to help patients re-experience pleasure in daily activities [113]. Although beyond the scope of this paper, the wide range of behaviourally-oriented therapies, methods of establishing rapport and ways of enhancing generalisation of in-therapy cognitive changes to real-world behavioural change is recognised here. For the purposes of the major argument being proposed (i.e., that inclusion of neurological phenomena within those behavioural frameworks will enable therapists to understand how depression occurs), this wide range of approaches is subsumed under the term "behavioural therapies".

If accentuated, the first of these therapeutic strategies (i.e., therapeutic rapport) can provide a powerful one-on-one interpersonal rapport experience for the depressed person, and may reduce hypercortisolaemia [107]. Support for this hypothesis comes from the finding that some behavioural therapies reduce "waking cortisol" [114] which is significantly associated with depression [115]. An analogy may be drawn between the hypercortisolemia observed in depression and that brought about by a lack of maternal stimulation and care given to neonates who consequently develop HPA-axis hyperactivation [116] and are vulnerable to early-onset depression during adolescence (although the potential for reversal of this is shown by hippocampal neurogenesis when maternal nurturance is reinstated [117]). Similarly, the intense verbal support and attention that is provided during psychological therapies may present a parallel nurturing experience for depressed patients who may previously have lost most sources of social reinforcement [107].

The second major strategy of behaviour therapies is that of behavioural activation, or encouraging the depressed person to engage in behaviours that will bring more positive social and personal enjoyment [113]. By replacing social-withdrawal behaviours with social-engagement behaviours, the previously-uncontrollable environmental stressor that instigated hypercortisolaemia (and its consequences to the PFC, hippocampus and amygdala that worked to help the depressed individual withdraw from their threatening environment), may become less important to the person facing it and therefore reduce the need for the accompanying (and functional) neurobiological sequence of withdrawal events that has been described above.

However, these neurobiological effects of behaviour therapies are valuable but often occur without the awareness of behaviour therapists. Based upon the links described above between environmental stressors, neurobiological sequelae, and overt behaviour, several other suggestions may be made so that behaviour therapists might actively incorporate neurobiological data into behaviourally-focussed therapies for depression. First, as well as standard psychological assessment and background information, induction to therapy for depression might include blood or saliva tests for hypercortisolaemia as an indicator of prolonged HPA activation that may lead to organic changes to the PFC, hippocampus and amygdala. Although more invasive, functional MRI assessments of those sections of the depressed patient's brain might also inform initial assessment and provide a baseline for evaluation after treatment. Second, although considered to be an important and existing aspect of those therapies, the level of rapport and support engendered in behavioural therapies should receive major attention because intense rapport has been shown to decrease cortisol (as described above). Therefore, this aspect of the patient-therapist interaction should be more than the superficial friendliness that is part of common professional demeanour [118-120]. Third, many depressed patients may experience depression following a tragic event such as death of a loved one, job loss, or a major personal injury that inhibits previously-available activities. Although the DSM-IV-TR states that depression arising from some of these events is actually "understandable intense sadness" rather than clinical depression [37], patients' feelings are real to them and may be experienced by them as depression. By having the functional value of the neurobiological events that are detailed in this paper explained to them during therapy, depressed patients may accept that their "depression" (i.e., social withdrawal) is functional, and thus indicative of their overall adaptability to major loss rather than of mental illness. Fourth, although the behavioural activation aspect of therapy (i.e., via homework in the real world) is a significant factor in patient change under those therapeutic approaches [113], emphasis on the beneficial effects of those activities that are designed to help patients expose themselves to multiple sources of distraction and social nurturance might be augmented by explanations of how they can potentially re-establish normal HPA axis activation, reduce serum cortisol levels, renew PFC and hippocampal neurogenesis, and re-establish the "balance" between emotional and rational decision-making about environmental stress. Fifth, the use of behavioural coaching may be beneficial for clients who are currently unable to instigate self-helping behaviour that enhances their social reinforcers.

## Conclusion

Although a wealth of research data support the use of behavioural models of depressive behaviour and behavioural therapies for treatment of depression, particularly those which emphasise a functional analysis component, the neurobiological underpinnings of the onset of depressive behaviour and its adaptive consequences for the person exhibiting it (and, consequentially, behavioural therapy approaches for treatment of depression) have not been joined in a comprehensive model. Built upon the notions of sickness behaviour as providing a benefit to the infected organism, the concomitant neurobiological alterations to structure and function of various brain regions (principally the PFC, hippocampus and amygdala) that accompany elevated HPA-axis activation following the onset of uncontrollable and highly aversive environmental stressors, may be seen to link observable behaviour with brain function to provide a more comprehensive model of depression. Further exploration of this model via monitoring of brain function during depressive experiences and therapy may help provide more detail for this model and move it towards greater completeness.

## Competing interests

**Financial competing interests**: The author(s) declare that they have no competing financial interests.

**Non-financial competing interests**: The author(s) declare that they have no competing non-financial interests.

## Authors' contributions

CS conceived the concept and designed the overall paper. He also wrote the sections on neurobiology of depression and about the ways in which the HPA axis links genes, environmental stress and depressive behaviour. VB wrote the section regarding behavioural therapies and functional analysis in depression, and also how the behavioural-brain model could be used in therapy. Both authors approved the final ms.

CS.

Both authors read and approved of the complete ms.

## Authors' information

VB is Associate Professor of Clinical Psychology and Behaviour Management, a State-Registered Clinical Psychologist, and has interests in behavioural and functional analysis of therapies for depression.

CS is a Professor of Clinical Neuroscience, with interests in the biological underpinnings of depression and treatment modalities.
